# Correlation between hemolytic activity, cytotoxicity and systemic in vivo toxicity of synthetic antimicrobial peptides

**DOI:** 10.1038/s41598-020-69995-9

**Published:** 2020-08-06

**Authors:** Ines Greco, Natalia Molchanova, Elin Holmedal, Håvard Jenssen, Bernard D. Hummel, Jeffrey L. Watts, Joakim Håkansson, Paul R. Hansen, Johan Svenson

**Affiliations:** 1 Department of Drug Design and Pharmacology, Faculty of Health and Medical Sciences, University of Copenhagen, Universitetsparken 2, 2100 Copenhagen, Denmark; 2 Department of Science and Environment, Roskilde University, 4000 Roskilde, Denmark; 3 Department of Chemistry, Biomaterial & Textile, RISE Research Institutes of Sweden, Box 857, 501 15 Borås, Sweden; 4 Zoetis Inc., 333 Portage St, Kalamazoo, MI 49007 USA; 5 Present Address: Department of Food Science, Faculty of Science, University of Copenhagen, Rolighedsvej 26, 1958 Frederiksberg, Denmark; 6 Present Address: The Molecular Foundry, Lawrence Berkeley National Laboratory, Berkeley, CA USA; 7 Present Address: Cawthron Institute, 98 Halifax Street East, Nelson, 7010 New Zealand; 8 Department of Laboratory Medicine, Institute of Biomedicine, University of Gothenburg, Box 440, 405 30 Gothenburg, Sweden

**Keywords:** Drug safety, Medicinal chemistry, Pharmacology, Toxicology, Drug development, Infectious diseases, Drug discovery, Medical research

## Abstract

The use of non-standard toxicity models is a hurdle in the early development of antimicrobial peptides towards clinical applications. Herein we report an extensive in vitro and in vivo toxicity study of a library of 24 peptide-based antimicrobials with narrow spectrum activity towards veterinary pathogens. The haemolytic activity of the compounds was evaluated against four different species and the relative sensitivity against the compounds was highest for canine erythrocytes, intermediate for rat and human cells and lowest for bovine cells. Selected peptides were additionally evaluated against HeLa, HaCaT and HepG2 cells which showed increased stability towards the peptides. Therapeutic indexes of 50–500 suggest significant cellular selectivity in comparison to bacterial cells. Three peptides were administered to rats in intravenous acute dose toxicity studies up to 2–8 × MIC. None of the injected compounds induced any systemic toxic effects in vivo at the concentrations employed illustrating that the correlation between the different assays is not obvious. This work sheds light on the in vitro and in vivo toxicity of this class of promising compounds and provides insights into the relationship between the different toxicity models often employed in different manners to evaluate the toxicity of novel bioactive compounds in general.

## Introduction

Antimicrobial peptides (AMPs) have attracted great interest in clinical research over the past three decades as potential therapeutic agents against multidrug resistant bacteria. AMPs are produced by most living organisms and they act as host defense peptides by providing a rapid first line of defense against pathogens^1,2^. They come in a variety of sizes and shapes but they are generally cationic and consist of less than 50 amino acids with antimicrobial activities in the low micromolar range^1,3^. The discovery that the natural peptides often can be significantly simplified with maintained biological activities makes them plausible candidates for the development of antimicrobials^4–7^. Currently, 36 antimicrobial peptides are undergoing preclinical and clinical phase^8^ and almost 10,000 papers have been published in 2019 on AMPs. While several natural lipo- and glycopeptides, e.g. colistin and vancomycin, have been approved by the Food and Drug Administration (FDA) as antibiotics, AMPs have yet to make a significant impact on the drug market despite often being heralded as a promising option for the future treatment of drug resistant bacterial and fungal infections^9^. AMPs are inherently not metabolically stable and their short half-lives and poor oral bioavailability, combined with potential toxic side effects such as haemolytic activity is hampering the clinical development^4,6^. One of the main advantages of AMPs as potential candidate drugs is their pronounced selectivity for bacterial and fungal cells over eukaryotic cells^3,10^. Hydrophobicity, charge and secondary structure are the main physicochemical driving forces determining the interactions between AMPs and their targets, which are mainly bacterial membranes^3,11,12^. Being AMPs and generally positively charged, their cellular selectivity is due to the high affinity to the negatively-charged lipid components of the bacterial membrane^10,11^. The outer layer of the cytoplasmic membrane of mammalian cells is, on the other hand, neutral and consists of mainly zwitterionic phospholipids and cholesterol, the latter has been suggested to further stabilise the membranes to the action of AMPs^3,13,14^. The initial interaction generally occurs through a complex interplay of phenomena including electrostatic and hydrophobic interaction with the bacterial membrane^9^, self-assembly and conformational transitions and induces cell death at sufficiently high concentrations^10^. Many models and modes of action have been described for membrane disruption by AMPs but no single general mechanism can be applied to explain the antimicrobial effects of all cationic AMPs. Furthermore, cell-penetrating AMPs targeting intracellular components are also known, adding a second layer of complexity to the mode of action of these peptides^5,10,15^. While the cellular selectivity provides the natural AMPs with a crucial host advantage in their role in the innate defense, it can become a challenge to maintain a sufficiently large therapeutic index over non-target normal host cells when preparing AMP-based therapeutic leads^12,16,17^. Several natural and synthetically improved AMPs also display ability to disrupt mammalian cells, thereby exerting an unwanted collateral toxicity, despite the difference in cellular membrane composition^16–18^.

Thus, an antimicrobial AMP drug candidate needs to show low or no toxicity towards mammalian cells to advance in the clinical process^19,20^. Over the past few years, a concern has been raised about the lack of a harmonised or standardised methodology for the assessment of toxic and haemolytic activity of AMPs^19,20^ and on how, quite often, in vitro toxicity data are not verified in vivo. In the majority of the literature reports on novel active AMPs, different concentration ranges and slight variations in the methods are adopted and thus the degree of haemolysis being reported as “safe” varies greatly between studies, without an actual in vivo assessment. As a result the comparison is often very difficult and makes conclusions potentially inaccurate.

Haemolysis represent the most commonly employed initial toxicity assessment. Although human erythrocytes is the most frequent choice for the preliminary in vitro testing of the haemolytic activity of AMPs intended for human use, it is not infrequent that erythrocytes sourced from other species are employed in the early screening phase. Several examples are available in the literature where AMPs targeting standard bacterial strains or human pathogens are tested against red blood cells collected from rodents^21–24^, or other mammalian species^25–27^. In addition, AMPs targeting veterinary pathogens are often tested for their haemolytic activity against human red blood cells^28,29^. This is generally accepted in the scientific community, but it may not provide an accurate assessment. In fact, very few studies comparing the susceptibility of AMPs on erythrocytes of different species^30^ or the efficacy of different blood media to assess haemolytic activity^19^ are published. This overlooked difference can provide additional ambiguity when comparing haemolytic results between compounds tested in different laboratories. For example, in a study by Rathinakumar et al.^31^, sheep and human erythrocytes show different haemolysis levels when exposed to AMPs (0.5, 5 and 15 µM), where sheep erythrocytes prove to be more resistant than human red blood cells^31^. Similar differences in haemolysis between bovine and human blood were reported by Lee et al.^32^ and Belokoneva and co-workers^30^. These preliminary studies indicate that the differences between mammalian erythrocyte sensitivity towards AMPs can be substantial and that the choice of potentially misleading toxicity controls can easily adversely affect the outcome or conclusions of a study^33^. There is thus an unmet need to clarify the extent of these inter-species variations on haemolytic activity of AMPs.

Efforts are made to design or optimise AMP structures with the highest therapeutic window but it is important to consider that both the antibacterial activity and haemolysis are governed by supplying sufficient hydrophobic bulk to disrupt the bacterial membrane, often making potency intimately coupled to haemolysis^16,19,34^. A strategy to overcome several of the general hurdles associated with peptide-based drugs is to incorporate unnatural amino acids^4,35,36^. These analogues have been shown to offer simple synthetic means towards increased antibacterial activity^36,37^, enzymatic stability^37–39^, uptake^40^ and protein binding^41^. Our previous studies on AMPs and related cationic peptoids have illustrated that incorporation of analogues of both cationic and hydrophobic amino acids allows the design of stable and highly efficient AMPs^37^. These unnatural residues can also be tools for controlling the therapeutic index of the final compounds^16^.

**Figure 1:**
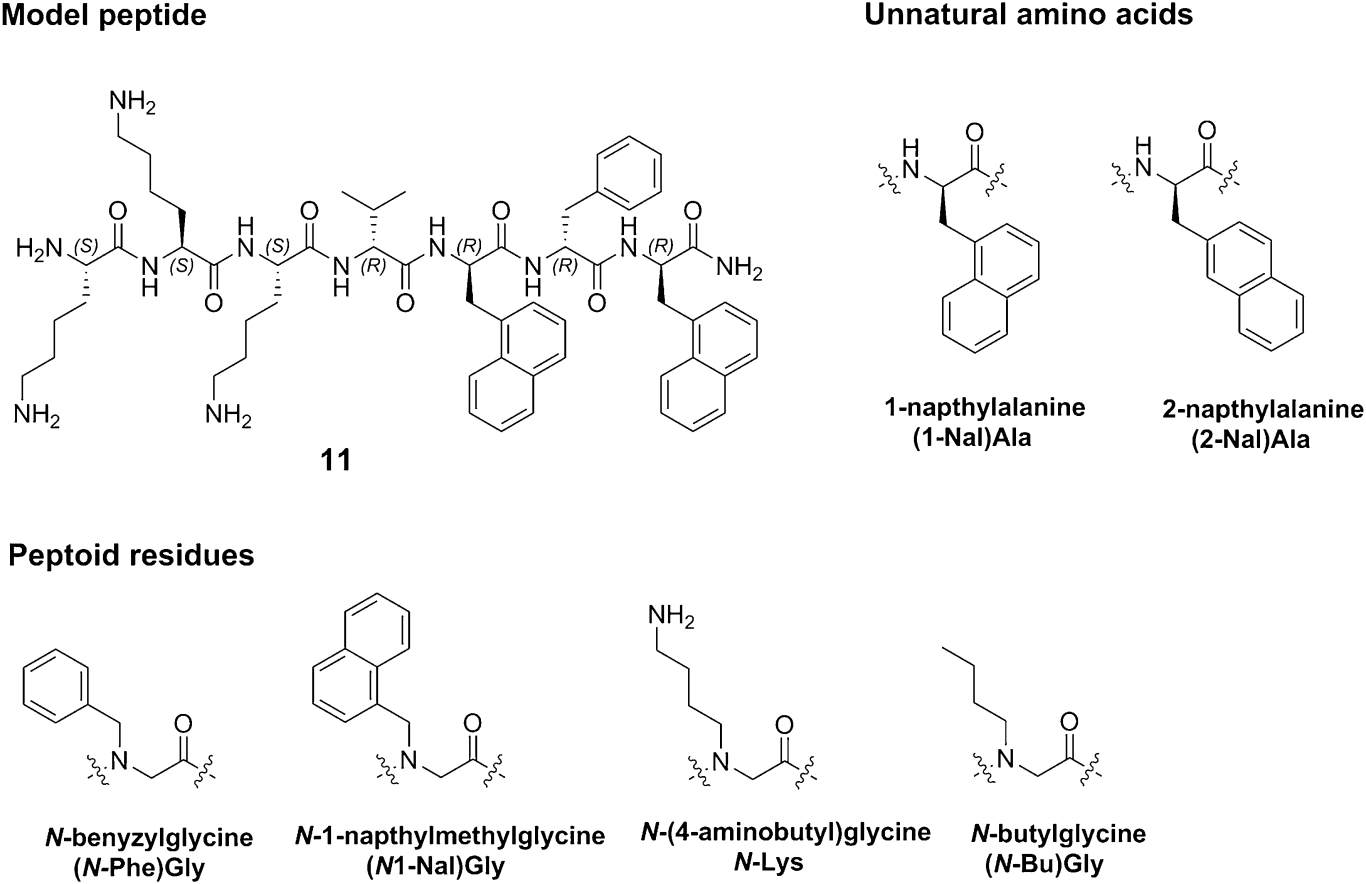
Structure of model peptide **11** and the structure of the unnatural and peptoid residues incorporated in the peptide library.

With these considerations in mind, our present study aims to be a step towards filling the existing knowledge gap between in vitro toxicity screening and effect observed in vivo by providing a toxicity profile for a series of promising narrow spectrum AMPs incorporating unnatural amino acids and peptoid elements^29^ in an attempt to assess the correlation between haemolytic activity, intra-species variation, cytotoxicity and in vivo data (displayed in Fig. 1 and Table 1 and tabulated in Table S1-S4). The peptide library evaluated contain promising narrow-spectrum antimicrobial agents towards integumentary infections and they range from highly active to inactive providing a relevant spread in physicochemical properties^29^. The study includes a comparative study of the haemolytic activity between human, dog, rat and bovine donors. In addition, the cytotoxic activity against non-target HeLa, HaCaT and HepG2 cells is also performed followed by an in vivo toxicity dose ranging study in rats including establishment of the haemolytic activity in vivo and observation of other potential adverse physiological effects.

**Table 1:** List of compounds used and their antimicrobial activity^29^.

ID	Sequence	R.t^a^	*S.p.*^b^	*S.a.*^c^	*P.a.*^d^
1	Lys-Lys-Leu-Lys-(2-Nal)Ala-Phe-(2-Nal-)Ala	17.19	8	16	32
2	(*N*1-Nal)Gly-(*N-*Phe)Gly-(*N*1-Nal)Gly-(*N-*Bu)Gly-Lys-Lys-Lys	17.43	8	16	32
3	**(2-Nal)Ala-Phe-(2-Nal)Ala-**Lys-**Leu**-Lys-Lys	15.84	16	32–64	> 64
4	**(2-Nal)Ala-Phe-(2-Nal)Ala-Leu-**Lys-Lys-Lys	16.47	16	32	> 64
5	(1-Nal)Ala-Phe-(1-Nal)Ala-Leu-Lys-Lys-Lys	15.98	16	32	> 64
6	Lys-Lys-Leu-Lys-(1-Nal)Ala-Phe-(1-Nal)Ala	16.96	8	32	8
7	Lys-Lys-Lys-Leu-(1-Nal)Ala-Phe(1-Nal)Ala	17.21	8	16	16
8	**(1-Nal)Ala-Phe-(1-Nal)Ala-**Lys-**Leu-**Lys-Lys	15.41	16	32–64	> 64
9	**(1-Nal)Ala-Phe-(1-Nal)Ala-Leu-**Lys-Lys-Lys	16.11	16	32	32
10	Lys-Lys-**Leu-**Lys-**(1-Nal)Ala-Phe-(1-Nal)Ala**	16.81	4–8	16	16–32
11	Lys-Lys-Lys-**Leu-(1-Nal)Ala-Phe-(1-Nal)Ala**	17.52	2	8	8
12	Lys-Lys-Lys-**Leu-(2-Nal)Ala-Phe-(2-Nal)Ala**	17.70	2–4	8–16	32
13	(2-Nal)Ala-Phe-(2-Nal)Ala-Lys-Leu-Lys-Lys	16.31	4–8	16	64
14	Lys-Lys-(*N-*Bu)Gly-Lys-(*N*1-Nal)Gly-(*N-*Phe)Gly-(*N*1-Nal)Gly	17.48	4	8–16	32
15	Lys-Lys-Lys-(*N-*Bu)Gly-(*N*1-Nal)Gly-(*N-*Phe)Gly-(*N*1-Nal)Gly	17.89	4	16	32
16	(1-Nal)Ala-Phe-(1-Nal)Ala-Lys-Leu-Lys-Lys	16.20	8–16	16–32	64
17	Lys-Lys-Lys-Leu-(2-Nal)Ala-Tyr-(2-Nal)Ala	16.62	16	> 64	> 64
18	Lys-Lys-Lys-Nle-(2-Nal)Ala-Phe-(2-Nal)Ala	17.46	2–4	32–64	8
19	Lys-Lys-Lys-Leu(1-Nal)Ala-Tyr-(1-Nal)Ala	17.36	n.t.^e^	> 64	> 64
20	Lys-Lys-Lys-Nle-(1-Nal)Ala-Phe-(1-Nal)Ala	16.55	16–32	> 64	16
21	Lys-Lys-Lys-**Leu-(1-Nal)Ala-Tyr-(1-Nal)Ala**	16.70	2–4	32–64	8
22	Lys-Lys-Lys-Nle-(2-Nal)Ala-Tyr-(2-Nal)Ala	16.67	2–4	64	64
23	*N*-Lys-*N*-Lys-*N*-Lys-Leu-(2-Nal)Ala-Phe-(2-Nal)Ala	16.98	2–4	32	32
24	Lys-Lys-Lys-**Leu-(2-Nal)Ala-Tyr-(2-Nal)Ala**	17.67	4–8	16–32	32

d-residues denoted in bold.

^a^Retention time (min).

^b^MIC (µg/mL) against *Staphylococcus pseudintermedius.*

^c^MIC (µg/mL) against *Staphylococcus aureus.*

^d^MIC (µg/mL) against *Pseudomonas aeruginosa.*

^e^Not tested.

## Results and discussion

### Haemolytic activity

**Figure 2:**
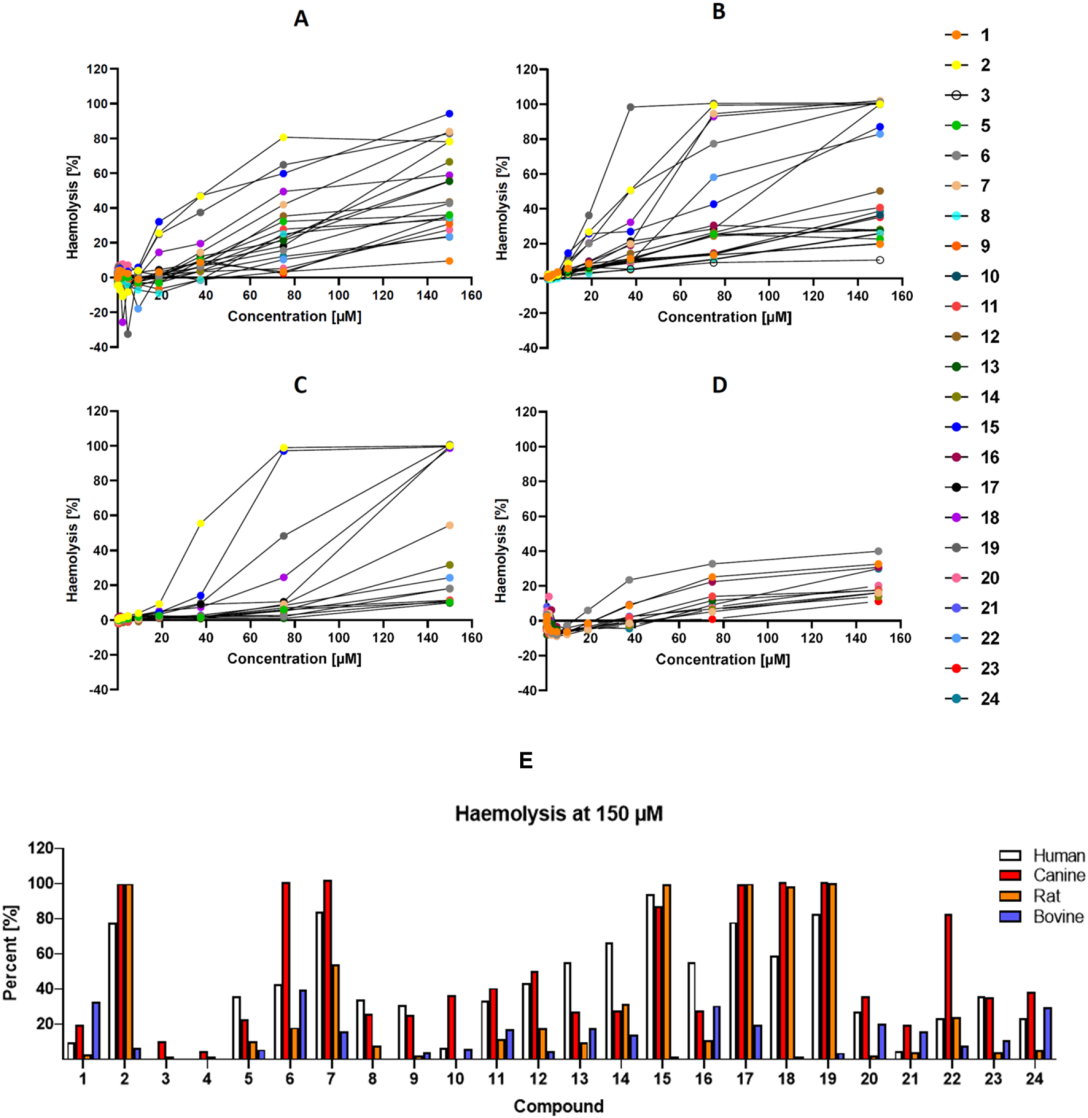
Haemolytic effect of the peptides. Upper images (**A**–**D**): Dose–response curves for the haemolytic activity of 24 structurally related peptide-based compounds in human (**A**), canine (**B**), rat (**C**) and bovine (**D**) erythrocytes, with concentrations ranging from 0.15 to 150 µM. Compounds with a haemolysis < 8% have been excluded from the graphs for clarity purposes. Lower image (**E**) illustrates the haemolysis at 150 µM against rat, canine, bovine and human erythrocytes.

The haemolytic activity of the 24 structurally related peptides (Table 1) was evaluated in erythrocytes from four different species (human, canine, bovine and rat) employing standard methodology. The subsequent release of haemoglobin was used to assess haemolytic activity as function of peptide and peptidomimetic concentration, with concentrations ranging from 0.15 to 150 µM (Presented in whole in Supplementary information Table S1–S4). The resulting dose–response curves are shown in Fig. 2.

The erythrocyte is often employed as model of mammalian cell membranes and the composition of the inner and outer leaflet has been studied in great detail for a range of mammalian species^19^. The ease associated with isolating erythrocytes makes the haemolytic activity assay a versatile tool for rapid initial toxicity assessment^42^ and it is thus often included in studies of membrane active antimicrobial agents and novel xenobiotics^43^. The membrane of the erythrocyte is composed of proteins and lipids, bound by non-covalent interactions, and glycoproteins, in compositions that vary between species^44^. Transmembrane proteins regulate the ion flow, mechanical properties and cell support and this can have a big effect on the cellular sensitivity to toxic compounds^43^. The protein composition and membrane organisation can vary significantly between species^45^, as well as the presence of aquaporins^46^, and the abundance of Na^+^/K^+^-ATPase^47^ which can account for differences in water permeability, diffusion and erythrocyte osmotic fragility^48^.

The main lipids of the dynamic erythrocyte membrane are phospholipids, followed by neutral lipids (mainly cholesterol) and glycosphingolipids^49^. The lipid distribution in erythrocytes varies between mammalian species^50^ with the cholesterol content being stable at approximately 25% of the total membrane lipids^51^, and the differences mainly seen in phospholipid distribution and content^44^. The membrane phospholipids can be divided into choline phospholipids (CPs, phosphatidylcholine and sphingomyelin), acidic phospholipids (APs, phosphatidylserine, phosphatidylinositol and phosphatidic acid) and phosphatidylethanolamine (PE)^52^. The individual phospholipids are unevenly distributed between the inner and outer leaflet of the erythrocyte membrane with the bulk of the outer leaflet exposing the CPs and PE^53^. This contributes to the neutral surface of the outer mammalian membrane which plays a key role in distinction from the net anionic bacterial membranes which is crucial for the cellular selectivity inherent in native innate AMPs and synthetics mimics^17^. The composition of the erythrocyte leaflets has been studied in detail for several mammalian species and is seemingly similar (Table 2) with the major exception being that the CP fraction for the bovine erythrocyte membrane is mainly composed of sphingomyelin (91.3%) as compared to others (45.8% of total CPs in humans)^54^.

**Table 2:** Overall phospholipid head group class composition (%) of the erythrocytes from the mammals included in the current study^a^.

Phospholipid	Erythrocytes			
	Human	Bovine	Canine	Rat
CPs	55.8	52.5	59.5	59.0
APs	16.6	15.4	18.1	16.0
PE	27.6	32.4	22.4	25.0

^a^Compositional data from Virtanen et al.^53^.

**Figure 3:**
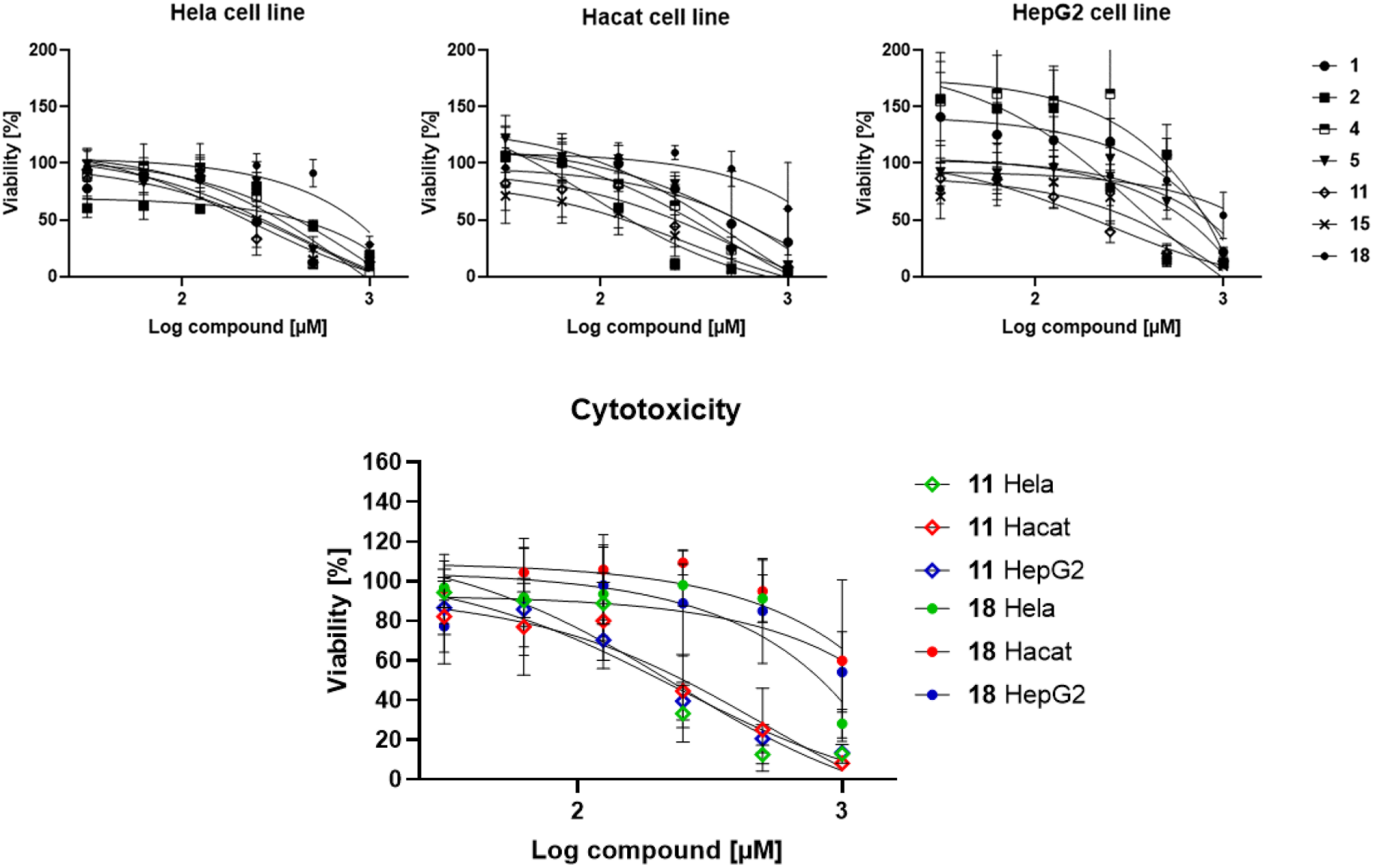
Upper row: effects of selected peptides on the viability of HeLa, HaCaT and HepG2 cells. Lower row; dose–response behavior of compounds **11** and **18** illustration differences in cellular response.

The susceptibility of erythrocytes to lysis by extracellular compounds depends on multiple factors, including species, assay conditions and buffers^43^. As shown in Figs. 2 and 3, the peptides display ranging activities towards the erythrocytes with some peptides like **7**, **15**, **18** and **19** being highly haemolytic (near total haemolysis at 80 µM against human, rat and dog erythrocytes). The majority of the peptides, however, display haemolysis < 40% at 150 µM (presented in Supplementary information Table S1–S4). The canine erythrocytes show the highest sensitivity, while human and rat cells are intermediate. The bovine erythrocytes, are generally less subject to lysis, with only 12 of the compounds showing haemolytic activity > 8% at 150 µM. Compound **6** is the most potent displaying 39.9% haemolysis in bovine erythrocytes at the highest concentration tested, which is in contrast to the other species and also to the physicochemical properties of the peptides. Overall, these results correlate with our previously reported haemolytic activities against human erythrocytes in which **6**, **7** and **18** were observed to display high in vitro haemolytic activity^29^.

The haemolytic activity against human, dog and rat is correlated to peptide hydrophobicity, with inactive compounds such as **3**–**5** and **8** and **9** also being the most polar ones in the study. These compounds were also shown in our previous study to display no haemolytic activity against human erythrocytes^29^. More haemolytic compounds such as **6**, **11**, **15**, **18** and **19** are also generally more hydrophobic, a property also evident in the antimicrobial effect of this type of compounds which is driven by both charge and hydrophobicity^17^. The more polar peptides display little or no haemolytic activity and they are also the least potent antimicrobials in the study. All the peptides are composed of the same building blocks and it is clear that  the selected sequences allow them to adopt more reactive solution conformers. The ability to form amphipathic solution structures has previously been linked to an enhanced antimicrobial activity for AMPs but also to increased haemolytic activity once a threshold hydrophobicity has been reached^12,55^. All compounds of the present study incorporate unnatural residues and selected ones include D-stereoisomers. The 1- and 2-napthylalanine residues are present in the majority of the compounds to provide hydrophobic bulk, and they do not seem to specifically display an effect on the haemolytic activity. The inclusion of D-stereoisomers has no profound effect on the antimicrobial activity, but generally results in a lowered haemolysis, often significantly. For example comparing **6** (all L) with **10** (mixed) and **13** (all L) with **3** (mixed) reveals the large difference in haemolysis induced by inclusion of the D-stereoisomers in **3** and **10**. This could indicate that the haemolytic target relies on interactions that are hampered by mixing of the residues^55^. Including mixed residues in AMPs have previously been shown to prevent the formation of optimised amphiphilic solution structures resulting in lowered biological activities^56^, potentially by altering the peptide membrane localization^57^. The incorporation of D-residues in the structure of AMPs has been related to different mechanisms of action towards bacterial cells^58^ and to different affinity towards bacterial peptidoglycan^59^. It is likely that the inclusion of D-residues here limits the interactions between the compounds and targets on or in the erythrocyte membrane. In contrast, other studies have indicated that mixed stereochemistries can be used to increase bioactivity or reduce toxicity of related cationic synthetic AMPs^60–62^ and these effect are clearly sequence dependent. In addition, the three peptoids **2**, **14** and **15** all contain *N*-butylglycine, *N*-1-naphthylmethylglycine and *N*-benzylglycine and this appears to generate more lytic compounds as these three peptoids are amongst the top five most haemolytic compounds against human erythrocytes. Compound **23**, containing *N*-(4-aminobutyl)glycine, is not highly lytic but lacks a non-peptoid reference compound for comparison. Cytosolic proteases from human erythrocytes have been shown to be able to degrade AMPs but no data from the current study indicate that this plays a prominent role for the erythrocyte stability against these compounds under the given experimental conditions^63^.

For the bovine erythrocytes it is seemingly different as the more hydrophobic peptides (**12**, **15** and **18)** in fact are nearly inactive even at 150 µM, while none of the evaluated compounds are able to induce total haemolysis. No clear links between peptoid content or stereochemistry is evident either. Ruminant erythrocytes exhibit a specific lipid composition^54^ which has been related to differences in membrane permeability^64,65^ and this is clearly reflected in the current study. It has previously been shown that the haemolytic activity of a range of AMPs is strongly affected by the phosphatidyl-to-sphingomyelin ratio in both isolated cells and lipid bilayers models where a higher stability is seen for cells with a higher content of sphingomyelin^30^. Additionally, the high phosphatidyl-to-sphingomyelin ratio of ruminant cells has also been shown to grant them an increased stability to the effects of lytic snake poison^66^. Sphingomyelin is involved in the formation of stable detergent membrane raft fragments and plays a key role in regulating the dynamic properties of biological membranes, in particular membrane fluidity^67^. A lowered phosphatidyl-to-sphingomyelin ratio results in low membrane fluidity and appears to play a decisive role for the susceptibility towards membrane active peptides and peptoids^68^. In addition the bovine erythrocyte displays an increased stability towards enzymatic degradation (bee venom Plase A_2_)^54^ and a lowered glycerol permeability in comparison to the human equivalent (facilitated via Aquaporin 3)^46^, which are additional factors illustrating the increased resilience of the bovine equivalent.

### Cytotoxicity

Haemolysis is often employed as a rapid method for initial assessment of cellular toxicity^43^. Isolated and washed erythrocytes however represent vulnerable cells and the different methods and buffers employed can result in significantly ranging results for the same compound^19,69^. Employing additional studies on other cell types using standardised methods allow a more accurate evaluation of the cytotoxic capacity of a compound and represent a valuable addition to the in vitro assessment. For this purpose, seven compounds (**1**, **2**, **4**, **5**, **11**, **15** and **18)** were selected for cytotoxic evaluation, representing different levels of haemolytic activity, ranging from the inactive **4** to the highly haemolytic peptide **15**. Selected compounds were evaluated against three different human cell lines (HeLa, HaCaT and HepG2 cells) commonly employed as reference cells. The sensitivity of the cells to the different compounds is summarised in Fig. 3.

The results from the cytotoxicity studies indicate a generally higher tolerance exhibited by the HaCaT, HepG2, and HeLa cells for the tested compounds in comparison to human erythrocytes. This is in correlation with other AMP studies where both types of cytotoxic assessment are included^70^. The estimates of the viability at 150 µM compared to the % haemolytic activity at 150 µM (supplementary information Table S1) show that the more haemolytic compounds are less toxic against the HaCaT, HepG2, and HeLa cells. IC_50_ values could not be obtained for all the compounds (supplementary information Table S5) but the majority of the compounds reduced the viability down to 0% at the highest employed concentration (1 mM). Overall, it appears that the compounds display similar toxicities to the different cell types and the links between compound hydrophobicity and amino acid residue composition and stereochemistry are not as pronounced as seen against bacteria or erythrocytes. In comparison to the bacterial MICs it is nevertheless clear that several of the more effective antimicrobials display pronounced selectivity indexes (50–500) which is comparable to several other designed AMP mimics^71^.

### Dose-ranging toxicity study in rat

To further address the actual correlation of the in vitro observations to an in vivo situation, three compounds were selected for animal studies. In vivo studies allow evaluation of complex systemic toxicity not achievable using cellular assays. Peptides where chosen to include compounds with ranging antibacterial activity, residue composition, hydrophobicity and in vitro mammalian toxicity, deliberately excluding compounds too hydrophobic and too toxic (50% > lysis at 150 µM in vitro against rats and human). The in vivo doses were based on the solubility limit in PBS of the compounds where **11** required mild heating and sonication at the highest concentration while **4** was freely soluble. The experimental compound solubility correlated well with the observed analytical retention times and highlight large differences in solubility (Supporting information Figure S1) within a relatively narrow elution range. All the compounds were tested at the same concentrations 0, 0.01 and 1.0 mg/kg rat.

**Figure 4:**
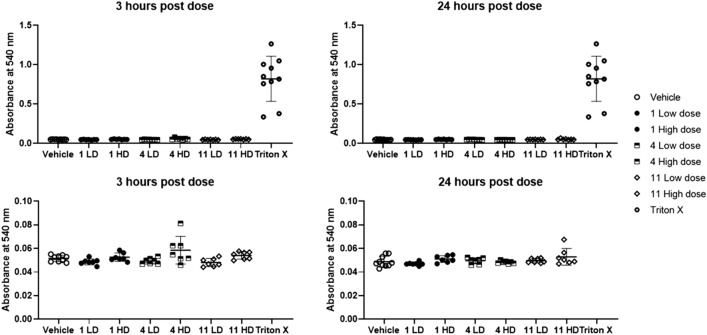
Variations in whole blood haemolysis for compounds **1**, **4** and **11** at low dose (LD) and high dose (HD) after 3 h and 24 h as compared to whole blood treated with Triton X. Bottom row shows close up of the whole blood haemolysis after 3 h and 24 h indication a small increase in release of haemoglobin after 3 h for peptide **4**.

All animals were injected with a single dose of 0.5 mL (2 mL/kg body weight) and it was slowly administered over 2 min and 10 s. Shorter cationic tripeptides^4^ and synthetic AMPs^72^ have previously been evaluated employing the intravenous route. It was an interesting observation that the animals survived the systemic tail vein injection seemingly unaffected. This observation is in contrast to related synAMPs investigated as intravenous antibiotics which induced acute toxicity and paralysis at higher concentrations^72^. Of the 59 animals included in our study, only two displayed clinical signs of discomfort after the initial 30 min of recovery from the anaesthesia following the injection. Both animals were injected with the high dose (1 mg/mL) of **4** and they initially displayed less mobility and reactivity to stimuli. In addition, no piloerection of the fur was seen at 4 h after the injection. At 8 h after the injections, no adverse effects were observed. The results from the haemolysis are presented in Fig. 4.

**Figure 5:**
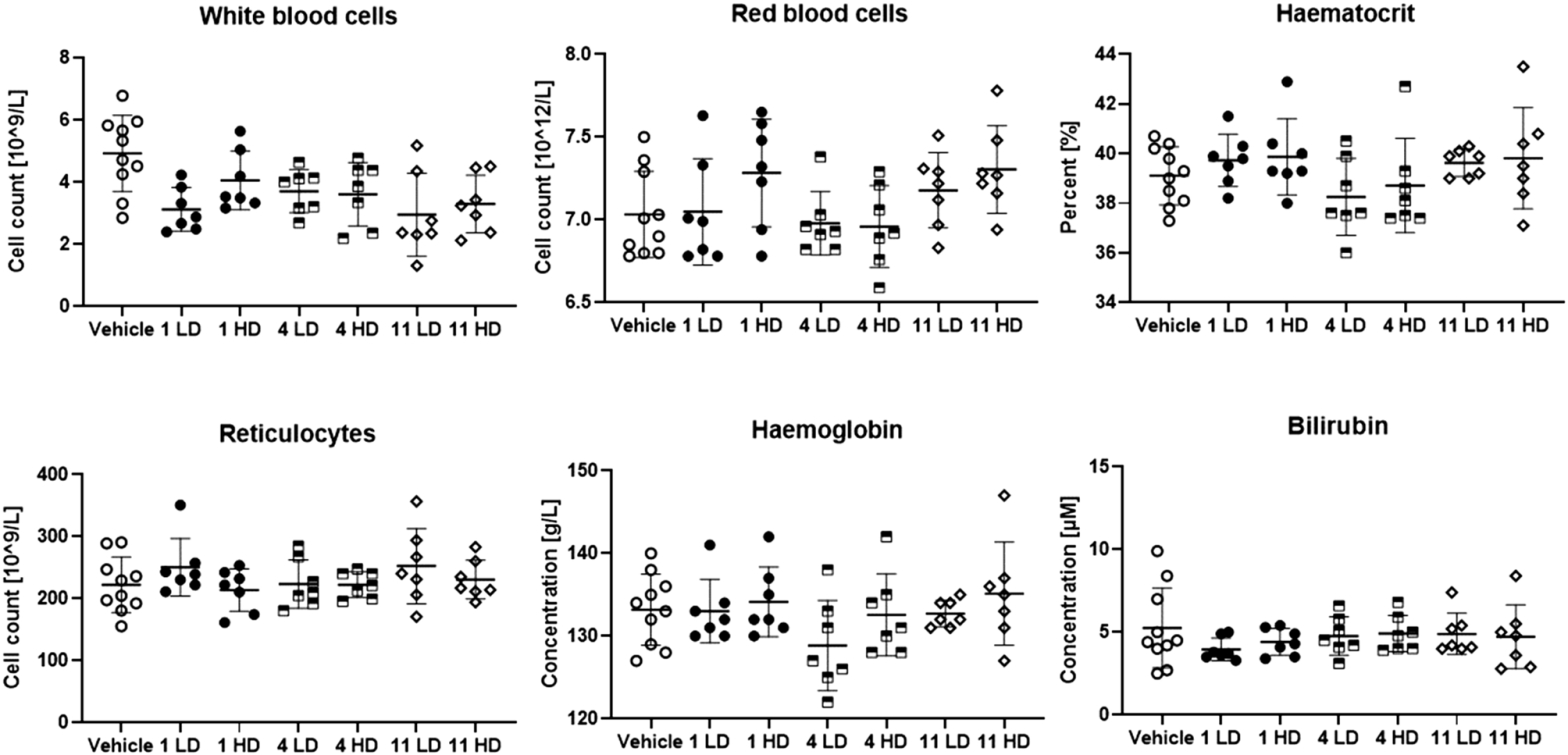
Haematologic parameters investigated for compounds **1**, **4** and **11** at low dose (LD) and high dose (HD).

No pronounced haemolysis was observed for any of the compounds at the investigated concentrations. However, for the high dose of **4**, at 3 h a slightly elevated release of haemoglobin was detected as shown in Fig. 4. The highest value was obtained from one of the animals initially displaying adverse clinical signs. Compound **11** display haemolysis in vitro at the higher concentration but this was not observed in vivo. It is believed that the slow and extended injection over 2 min is a contributing factor for avoiding a potential local toxic effect of the high concentration. The haematologic evaluation reveals additional insight into the effect of the injected peptides and can provide support for inflammatory responses^73,74^. A summary of the haematologic and plasma parameters are presented in Fig. 5.

**Figure 6:**
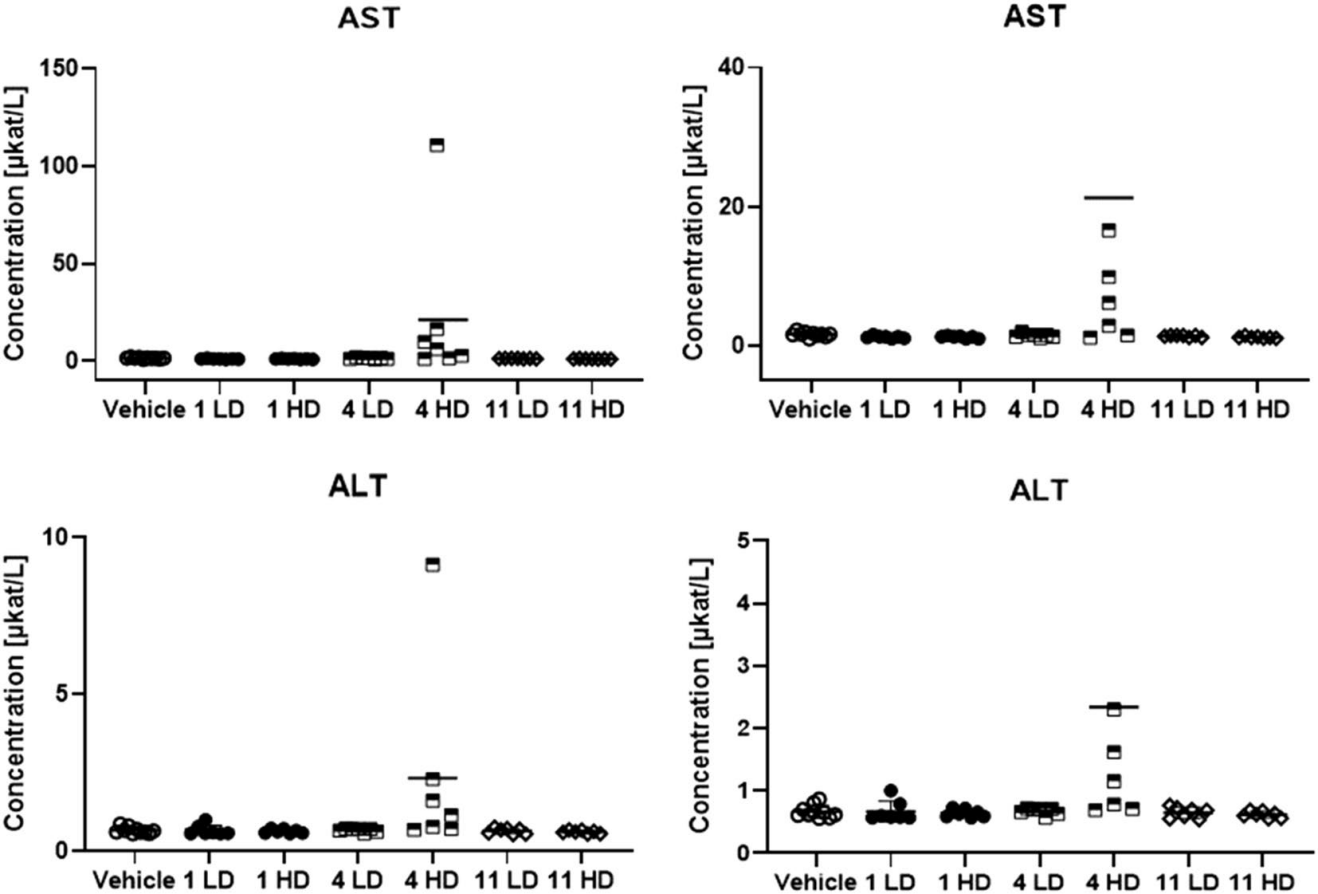
The concentrations of AST and ALT in response to injected peptides **1**, **4** and **11** at low dose (LD) and high dose (HD).

Most of the haematologic parameters suggest no direct reaction to the injection of the antibacterial peptides. No inflammatory response, such as for example an elevated white blood cell count, could be detected using these markers^74^. Further analysis of the plasma indicates no effect on the concentration of either urea, albumin or creatine (supplementary information Figure S2). Analysis of aspartate transaminase (AST) and alanine transaminase (ALT) concentration is an established method to monitor liver health and the AST/ALT ratio can be indicative of a range of conditions^75^. The effect of the injected peptides on AST and ALT are shown in Fig. 6.

A slight increase of both AST and ALT was seen for compound **4** at the high dose, while the other compounds result in no significant change in AST and ALT. The outlier again being the same animal in which the highest haemolytic activity was observed. This data suggests that compound **4** could display liver toxicity at elevated concentrations but that need to be further studied. The bilirubin concentration is also a marker of liver toxicity^76^ but no elevated concentration was observed for **4** (Fig. 5).

The pathological investigation revealed no major changes at the injection site. The abdominal and thoracic cavities were also normal, and no weight loss was recorded for the animals during the 24 h experiment. The tissues and major organs were dissected, weighed and macroscopically evaluated revealing no change in appearance or weight. No histological study was performed. Collectively, the in vivo studies illustrate no or low acute in vivo toxicity of the studied peptides at the investigated concentrations. A slow injection appeared to be a successful way for intravenous administration of this type of compounds without inducing injection-related adverse effects. None of the major blood markers were affected by the compounds. The high dose of **4** induces a minor increase in haemolysis and an upregulation of both AST and ALT which could be signs of systemic toxicity. If these two effects are connected or have different origins is unclear. The peptides were injected at concentration resulting in a plasma concentration near 15 µM, assuming a 17 mL blood volume for a 250 g rat^77^. This equals to 2–8 × MIC for **11** and can be regarded as a realistic clinical dose. At these concentrations, the compounds display no or low haemolysis in the in vitro assays towards rat erythrocytes which correlates well with the in vivo observations.

### Assay relationships

For natural and synthetic membrane-active antimicrobials, the haemolytic experiment is often employed as the standard way to indicate the therapeutic index of the prepared compounds and numerous scientific papers are indeed concluded “the lead compound displayed x % haemolysis at y µM and should thus be safe for human use”. We argue that such statements should be critically considered as they are often insufficiently backed up to provide an accurate prediction of an in vivo situation. One of the main challenges and limitations of the haemolysis experiment is the numerous ways it can be performed. This does not only generate ranging absolute values but it makes comparison between studies complicated. Already small changes such as choice of buffer or amount of DMSO^78^ potentially needed to generate a stock solution will generate different values. This is clearly evident for this type of membrane active compounds, as shown in our previous studies where the highly active AMP LTX109 display an EC_50_ 175 µM in phosphate buffered saline (PBS)^79^ while in non-buffered physiological saline the EC_50_ was approximately 75 µM^78^ against human erythrocytes. What this lysis really mean in an in vivo situation is also hard to assess as these experimental conditions are far away from the buffered and protein-rich environment encountered in full blood. In the case of LTX109, a closely related analogue was evaluated (EC_50_ 720 µg/mL in PBS)^79^ in vivo and was shown to be tolerated at single intravenous and intraperitoneal doses of 12–14 mg/kg (~ 175–200 µg/mL) which perhaps is unexpected^4^. These types of compounds are cationic and amphiphilic, and they are prone to rapidly associate with both major exogenic transport plasma proteins serum albumin and α-1 glycoprotein. Recent studies illustrate that several binding modes are available for short cationic AMPs^41,80,81^ to these carriers. Addition of serum albumin at physiological concentrations to a MIC-experiment effectively resulted in a tenfold reduction in potency illustrating the ability of albumin to soak up lipophilic AMPs^41^ and this is a parameter not accounted for in the haemolysis experiment.

The current study highlights the importance of establishing and considering the source of the erythrocytes when comparing EC_50_ values. While the membrane of the human erythrocyte is structurally similar to mammals such as dog, monkey, horse, rat, rabbit and mouse^53,82^ clear differences exist in comparison with ruminants such as cows, sheep and camel^30,54^. It is thus crucial to take this into account when evaluating haemolysis data and when comparing with published data. Our current study illustrates that the bovine erythrocytes display a pronounced resilience towards the hydrophobic and more antibacterial peptides and several of the most active bactericidal peptides are in fact inactive (< 8% haemolysis at 150 µM), which is in stark contrast to the sensitivity seen particularly against the rat and dog erythrocytes. Studies not taking this into account often report huge therapeutic indexes which can be misleading unless the erythrocyte origin is clear^83^.

A more standardised method to establish toxicity is using proliferation assays against different types of human cell lines. Results from these types of studies are often more readily comparable and the possibility to use the same cells from the same provider opens up for more reliable comparisons. Comparing IC_50_ with EC_50_ for AMPs generally results in similar observations as what we observe with increased IC_50_ values and an apparent elevated robustness against the AMPs^25^. Whether this in fact is an increased resilience of the cells or more a reflection of AMP inactivation by the presence of fetal bovine serum^84^ in the medium is often not discussed and it is important to realise that washed erythrocytes analysed in non-buffered conditions represent different experimental conditions when comparing toxicity data for AMPs.

The results from the cytotoxicity and haemolysis studies form the basis for compound selection for in vivo studies. The in vivo experiment is the final stage before potential studies on human subjects, and provides data on a plethora of functions and markers in addition to haemolysis and viability. The current study was focused on studying the in vivo effects of these promising peptides but also to probe the links between the in vitro experiment and the effect potentially observed in vivo. The in vivo experiments come with additional challenges and one being dosing. To reach relevant concentrations of AMPs in vivo it is often necessary to inject millimolar amounts of compounds and this limits the ability to include some of the more active antimicrobials as they display poor solubilities in PBS or saline which are preferred solvents tolerated well by the animals. Hence we were limited in our study and **11** was the most active peptide we could include in the in vivo studies with a solubility threshold at 1 mg/mL in PBS yielding a maximal serum concentration of 15 µM. None of the three injected compounds displayed any pronounced toxic effects in vivo and the only potentially adverse effects were seen for **4** where the high dose induced an increase of both AST and ALT. Peptide **4** is a compound which is non-haemolytic.

Collectively the results from the current study represents the first comprehensive toxicity study of a series of potent narrow spectrum antimicrobial peptides incorporating both unnatural amino acids and peptoid elements. The haemolytic activity and toxicity was shown to be linked to a range of physicochemical properties of the compounds and the membrane composition of the target cells. None of the three injected compounds displayed any pronounced toxic effects in vivo. As such, the study provides detailed insights into the in vitro and in vivo cytotoxicity of membrane active antimicrobials and their interspecies differences.

## Materials and methods

### Synthetic peptides and peptide-peptoid hybrids

A set of 24 synthetic 7-residue long cationic peptides and peptide-peptoid hybrids has been used in the current study. All compounds were prepared as peptide amides and isolated and tested as TFA salts. The compounds combine both unnatural amino acids and *N*-alkylglycine/peptoid residues as illustrated in Fig. 1. The antimicrobial activity against Gram-positive and Gram-negative bacteria and preliminary human haemolysis of the compounds has been recently reported^29^. The compounds were synthesised using Fmoc solid phase peptide or a combination of solid phase peptide synthesis and sub-monomer peptoid synthesis (**2**, **14**, **15** and **23**) as previously described^29^. The compounds and their antimicrobial activity are reported in Table 1.

### Haemolytic activity

Human and rat (Sprague–Dawley) blood samples were purchased by Bioreclamation IVT (Westbury, NY, USA), collected in tubes containing heparin as anticoagulant and stored at 4 °C before use. Dog and bovine whole blood was collected from in-house animals at Zoetis Animal Health, Kalamazoo, Michigan and stored at 4 °C in tubes containing heparin as anticoagulant. 10 mL of each whole blood sample were centrifuged at 1,500 × *g* for 5 min and the resulting plasma fraction was removed from the samples. The pellets were washed with an equal volume (10 mL) of saline, mixing by inversion. The centrifuging and washing steps were repeated 5 times. After the last washing step, a volume of PBS was added, and the resulting samples were diluted 1:10 with PBS to yield an RBC concentration of ~ 5 × 10^8^ RBC/mL. The assay was performed in a 384-well polypropylene microplate (Greiner 784201, Greiner Bio-One International, GMBH) through a Bravo Liquid Handling Platform 384 system (Agilent Technologies, Santa Clara, CA, USA) according to the previously published procedures^29^. The compounds were tested in serial 1:1 dilutions in PBS, with final concentrations ranging from 150 to 1.2 µM. Melittin was used as reference lytic peptide. 1% TritonX100 and PBS were used as positive and negative (diluent) controls, respectively. The final concentrations were obtained by adding to the wells 7 µL of each solution, melittin or PBS, followed by 63 µL of RBCs to each well. The plate was sealed, shaken on a plate shaker for 20 s, incubated for one hour at 37 °C. Finally, it was centrifuged at 1,500 × *g* for 5 min at room temperature. 60 µL of supernatants were removed from all plate wells via a Bravo 384 robot and transferred to a flat-bottomed 384-well plate (Greiner UV-Star #7810801, Greiner Bio-One International, GMBH). The plate was centrifuged at 1,000 *g* for 1 min to remove air bubbles and optical density was measured at 405 nm using a plate reader. Percentage of haemolysis was calculated with the following equation:

$$\% {\text{ haemolysis }} = \frac{{({\text{absorbance of test sample}}) \, - \, \left( {\text{ absorbance of diluent}} \right)}}{{\left( {\text{absorbance of positive control}} \right) \, - \, \left( {\text{absorbance of diluent}} \right)}} \times 100.$$

### Cytotoxicity

An immortalised human keratinocyte (HaCaT) cell line (Gift from David Gram Naym at Bispebjerg Hospital, Denmark), a human liver cancer cell line (HepG2; ATCC HB-8065) and a human epithelial cervical cancer cell line (HeLa; ATCC CCL-2) were cultured to ∼90% confluence after 21–25 h of growth under standard conditions (5% CO_2_ at 37 °C). Cells were cultured in Dulbecco’s Modified Eagle’s Medium supplemented with 10% (v/v) fetal bovine serum (FBS). All culture media were supplemented with penicillin (100 IU/mL) and streptomycin (100 μg/mL). All cell media and supplements were obtained from Sigma-Aldrich (St. Louis, MO, United States). The 96-well plates were supplied by Corning Costar (Sigma-Aldrich, Brøndby, Denmark). Cell viability assessment was performed on cell monolayers grown to ∼90% confluence in 96-well plates by using the MTS/PMS assay as previously described^85^. Briefly, the adhered cells were washed with PBS solution (37 °C) (ThermoFisher Scientific, Roskilde) and exposed for 1 h at 37 °C to 100 μL of peptides dissolved in the medium also used for culturing of the cell line (at concentrations in the range 0–1,000 μg/mL). The cells were subsequently washed twice with 37 °C PBS and then 100 μL of an MTS/PMS solution in media, consisting of 240 μg/mL MTS (Promega, Madison, WI, United States) and 2.4 mg/mL PMS (Promega, Madison, WI, United States), was added to the cells, which then were incubated for 1 h at 37 °C protected from light. A plate reader (SpectraMax i3X; Molecular devices, San Jose, CA) was used to measure the absorbance at 492 nm. The relative viability was calculated by using 0.2% (w/v) sodium dodecyl sulfate (SDS) as the positive control, while cells exposed to medium without test compound were used as the negative control. Data were obtained in three independent biological replicates performed on separate passages of cells and on separate days with a total number of six replicates and the mean is presented with standard deviation in the graphs.

### Dose-ranging toxicity study in rats

The animal experiments were performed after prior approval from the local Ethics Committee for Animal Studies at the Administrative Court of Appeals in Gothenburg, Sweden. Hence, all procedures related to the treatment and holding of animals used in this study were approved under Swedish legislation (Dnr. 1939–2018, Gothenburg region, Sweden). All national animal welfare requirements were followed, and the animal housing was environmentally enriched for best animal comfort. The guidelines from the EU directive covering the use of animals for scientific purposes (DIRECTIVE 2010/63/EU) was followed and all animals were treated according to national and EU welfare requirements, minimizing stress and pain. They were under appropriate care and surveillance by technical personnel and veterinarians.

The compounds were formulated in sterile PBS (tablets, Medicago, Sweden), sonicated and sterile filtered through a Millex-GV filter (0.22 µm pore size, Ø 13 mm, Merck) before use. All solutions displayed a pH near 7 at the concentrations prepared. 59 female, ~ 250 g WistarHan rats (Charles River, Italy) were used for the study. The animals were acclimatised for seven days prior to the injections with free access to feed and water. For the in vivo experiment, rats were given a single 0.5 mL intravenous tail vein injection under isoflurane anaesthesia and monitored for 24 h. Doses were 0 (vehicle control), 0.01 and 1.0 mg/kg rat). Clinical observations were performed at regular intervals during the 24 h period to assess potential haemolysis related events such as muscle cramps or reduced urination. Pilot animals were initially dosed with the increasing peptide doses and vehicle prior to larger dosing groups to mitigate the risk of dosing an entire dose level at a toxic level. Whole blood was collected from each animal after 3 h (tail vein) and 24 h (heart, terminal bleeding under isoflurane anaesthesia) and transferred to EDTA tubes (Microvette 500 LH, Sarstedt). While kept on ice, 200 µL of the blood was transferred to a 96 well plate which was spun at 1,000 × *g* for 10 min at 4 °C before the plasma supernatant was transferred to a new 96 well plate. The plasma was diluted with PBS (1:30 v/v) and the release of haemoglobin was assessed spectroscopically at 540 nm. Control animal plasma treated with 2% Triton-X served as positive haemolysis control. The 24 h terminal blood sample was collected via heart puncture transferred to LiHep tubes (Microvette 500 K3E, Sarstedt) and centrifuged at 1,500 × *g* for 10 min at 4 °C. A minimum of 300 µL of plasma was transferred to safeseal tubes (Biosphere SafeSeal) and subjected to an extensive haematologic evaluation at CaniLab-EquiLab AB (Halmstad, Sweden) and the mean is presented with standard deviation in the graphs for all analyses. After the 24 h blood sample, the inferior *vena cava* was cut for euthanization and the animals were investigated for macroscopic abnormalities to the injections site and the thoracic and the abdominal cavity. The major organs (liver, spleen, kidneys and thymus) were dissected out and saved in formalin for histological evaluation.

## Supplementary information

Supplementary file1
