# Supplementary material for: Correlation between hemolytic activity, cytotoxicity and systemic in vivo toxicity of synthetic antimicrobial peptides
Source: Sci Rep. 2020 Aug 6;10:13206. doi: 10.1038/s41598-020-69995-9 (PMC7414031; doi:10.1038/s41598-020-69995-9)
Supplement: Supplementary file 1 — Supplementary file1 [file 41598_2020_69995_MOESM1_ESM.pdf]

Supporting information

Interspecies-variation of haemolytic activity, cytotoxicity and *in vivo* systemic toxicity of synthetic antimicrobial peptides

Ines Greco, Natalia Molchanova, Elin Holmedal, Håvard Jenssen, Bernard D. Hummel, Jeffrey L. Watts, Joakim Håkansson, Paul R. Hansen, Johan Svenson[*]

List of contents **Page**

Table S1. Haemolysis data against human erythrocytes 2
Table S2. Haemolysis data against canine erythrocytes 3
Table S3. Haemolysis data against rat erythrocytes 4
Table S4. Haemolysis data against bovine erythrocytes 5
Table S5. Cytotoxicity against HeLa, HaCaT and HepG2 cells 7
Fig S1. Differential hydrophobicity of *in vivo* compounds 8
Fig S2. Plasma concentration of urea, albumin and creatine in response to injection of the peptides 9

Table S1. Haemolysis data against human erythrocytes

ID	Sequence	Haemolysis 150 µM (%)	IC_{10} (µM)	IC_{50} (µM)
**1**	Lys-Lys-Leu-Lys-(2-Nal)Ala-Phe-(2-Nal-)Ala	<8	>150	>150
**2**	(*N*1-Nal)Gly-(*N*-Phe)Gly-(*N*1-Nal)Gly-(*N*-Bu)Gly-Lys-Lys-Lys	78.1	11.2	40.0
**3**	**(2-Nal)Ala-Phe-(2-Nal)Ala-**Lys-**Leu-**Lys-Lys	<8	>150	>150
**4**	**(2-Nal)Ala-Phe-(2-Nal)Ala-Leu-**Lys-Ly-Lys	<8	>150	>150
**5**	(1-Nal)Ala-Phe-(1-Nal)Ala-Leu-Lys-Lys-Lys	36.0	35.3	>150
**6**	Lys-Lys-Leu-Lys-(1-Nal)Ala-Phe-(1-Nal)Ala	42.7	55.6	>150
**7**	Lys-Lys-Lys-Leu-(1-Nal)Ala-Phe(1-Nal)Ala	83.8	35.2	82.6
**8**	**(1-Nal)Ala-Phe-(1-Nal)Ala-**Lys-**Leu-**Lys-Lys	34.3	57.0	>150
**9**	**(1-Nal)Ala-Phe-(1-Nal)Ala-Leu-**Lys-Lys-Lys	30.8	104.1	>150
**10**	Lys-Lys-**Leu-**Lys-**(1-Nal)Ala-Phe-(1-Nal)Ala**	<8	>150	>150
**11**	Lys-Lys-Lys-**Leu-(1-Nal)Ala-Phe-(1-Nal)Ala**	33.3	32.8	>150
**12**	Lys-Lys-Lys-**Leu-(2-Nal)Ala-Phe-(2-Nal)Ala**	43.5	34.6	>150
**13**	(2-Nal)Ala-Phe-(2-Nal)Ala-Lys-Leu-Lys-Lys	55.3	44.2	136.5
**14**	Lys-Lys-(*N*-Bu)Gly-Lys-(*N*1-Nal)Gly-(*N*-Phe)Gly-(*N*1-Nal)Gly	66.4	54.0	117.6
**15**	Lys-Lys-Lys-(*N*-Bu)Gly-(*N*1-Nal)Gly-(*N*-Phe)Gly-(*N*1-Nal)Gly	94.2	8.4	41.7
**16**	(1-Nal)Ala-Phe-(1-Nal)Ala-Lys-Leu-Lys-Lys	55.5	47.4	133.6
**17**	Lys-Lys-Lys-Leu-(2-Nal)Ala-Tyr-(2-Nal)Ala	78.1	61.1	108.6
**18**	Lys-Lys-Lys-Nle-(2-Nal)Ala-Phe-(2-Nal)Ala	58.8	18.7	95.6
**19**	Lys-Lys-Lys-Leu(1-Nal)Ala-Tyr-(1-Nal)Ala	82.7	13.5	51.1
**20**	Lys-Lys-Lys-Nle-(1-Nal)Ala-Phe-(1-Nal)Ala	27.5	91.3	>150
**21**	Lys-Lys-Lys-**Leu-(1-Nal)Ala-Tyr-(1-Nal)Ala**	<8	>150	>150
**22**	Lys-Lys-Lys-Nle-(2-Nal)Ala-Tyr-(2-Nal)Ala	23.7	88.3	>150
**23**	*N*-Lys-*N*-Lys-*N*-Lys-Leu-(2-Nal)Ala-Phe-(2-Nal)Ala	35.9	98.7	>150
**24**	Lys-Lys-Lys-**Leu-(2-Nal)Ala-Tyr-(2-Nal)Ala**	23.4	76.72	>150

Table S2. Haemolysis data against canine erythrocytes

**ID**	**Sequence**	**Haemolysis 150 µM**	**IC_{10} (µM)**	**IC_{50} (µM)**
**1**	Lys-Lys-Leu-Lys-(2-Nal)Ala-Phe-(2-Nal-)Ala	19.7	34.2	>150
**2**	(*N*1-Nal)Gly-(*N*-Phe)Gly-(*N*1-Nal)Gly-(*N*-Bu)Gly-Lys-Lys-Lys	100.1	12.8	31.99
**3**	**(2-Nal)Ala-Phe-(2-Nal)Ala-**Lys-**Leu-**Lys-Lys	10.5	118.3	>150
**4**	**(2-Nal)Ala-Phe-(2-Nal)Ala-Leu-**Lys-Ly-Lys	<8	>150	>150
**5**	(1-Nal)Ala-Phe-(1-Nal)Ala-Leu-Lys-Lys-Lys	22.5	29.6	>150
**6**	Lys-Lys-Leu-Lys-(1-Nal)Ala-Phe-(1-Nal)Ala	101.2	12.2	37.0
**7**	Lys-Lys-Lys-Leu-(1-Nal)Ala-Phe(1-Nal)Ala	102.1	32.7	47.2
**8**	**(1-Nal)Ala-Phe-(1-Nal)Ala-**Lys-**Leu-**Lys-Lys	25.8	66.0	>150
**9**	**(1-Nal)Ala-Phe-(1-Nal)Ala-Leu-**Lys-Lys-Lys	25.5	65.0	>150
**10**	Lys-Lys-**Leu-**Lys-**(1-Nal)Ala-Phe-(1-Nal)Ala**	36.3	43.4	>150
**11**	Lys-Lys-Lys-**Leu-(1-Nal)Ala-Phe-(1-Nal)Ala**	40.7	30.4	>150
**12**	Lys-Lys-Lys-**Leu-(2-Nal)Ala-Phe-(2-Nal)Ala**	50.2	29.3	>150
**13**	(2-Nal)Ala-Phe-(2-Nal)Ala-Lys-Leu-Lys-Lys	27.4	27.1	>150
**14**	Lys-Lys-(*N*-Bu)Gly-Lys-(*N*1-Nal)Gly-(*N*-Phe)Gly-(*N*1-Nal)Gly	28.1	26.23	>150
**15**	Lys-Lys-Lys-(*N*-Bu)Gly-(*N*1-Nal)Gly-(*N*-Phe)Gly-(*N*1-Nal)Gly	87.1	11.6	65.7
**16**	(1-Nal)Ala-Phe-(1-Nal)Ala-Lys-Leu-Lys-Lys	27.7	16.4	>150
**17**	Lys-Lys-Lys-Leu-(2-Nal)Ala-Tyr-(2-Nal)Ala	100.0	59.6	87.0
**18**	Lys-Lys-Lys-Nle-(2-Nal)Ala-Phe-(2-Nal)Ala	100.7	21.4	42.7
**19**	Lys-Lys-Lys-Leu(1-Nal)Ala-Tyr-(1-Nal)Ala	100.8	13.2	20.8
**20**	Lys-Lys-Lys-Nle-(1-Nal)Ala-Phe-(1-Nal)Ala	36.0	45.8	>150
**21**	Lys-Lys-Lys-**Leu-(1-Nal)Ala-Tyr-(1-Nal)Ala**	19.9	48.2	>150
**22**	Lys-Lys-Lys-Nle-(2-Nal)Ala-Tyr-(2-Nal)Ala	83.0	30.9	71.2
**23**	*N*-Lys-*N*-Lys-*N*-Lys-Leu-(2-Nal)Ala-Phe-(2-Nal)Ala	35.1	43.3	>150
**24**	Lys-Lys-Lys-**Leu-(2-Nal)Ala-Tyr-(2-Nal)Ala**	38.5	45.0	>150

Table S3. Haemolysis data against rat erythrocytes

ID	Sequence	Haemolysis 150 µM	IC_{10} (µM)	IC_{50} (µM)
**1**	Lys-Lys-Leu-Lys-(2-Nal)Ala-Phe(2-Nal-)Ala	<8	>150	>150
**2**	(*N*1-Nal)Gly-(*N*-Phe)Gly-(*N*1-Nal)Gly-(*N*-Bu)Gly-Lys-Lys-Lys	100.0	20.3	35.1
**3**	**(2-Nal)Ala-Phe-(2-Nal)Ala-**Lys-**Leu-**Lys-Lys	<8	>150	>150
**4**	**(2-Nal)Ala-Phe-(2-Nal)Ala-Leu-**Lys-Ly-Lys	<8	>150	>150
**5**	(1-Nal)Ala-Phe-(1-Nal)Ala-Leu-Lys-Lys-Lys	10.2	147.7	>150
**6**	Lys-Lys-Leu-Lys-(1-Nal)Ala-Phe-(1-Nal)Ala	18.2	120.5	>150
**7**	Lys-Lys-Lys-Leu-(1-Nal)Ala-Phe(1-Nal)Ala	54.3	76.9	142.9
**8**	**(1-Nal)Ala-Phe-(1-Nal)Ala-**Lys-**Leu-**Lys-Lys	<8	>150	>150
**9**	**(1-Nal)Ala-Phe-(1-Nal)Ala-Leu-**Lys-Lys-Lys	<8	>150	>150
**10**	Lys-Lys-**Leu-**Lys-**(1-Nal)Ala-Phe-(1-Nal)Ala**	<8	>150	>150
**11**	Lys-Lys-Lys-**Leu-(1-Nal)Ala-Phe-(1-Nal)Ala**	11.3	128.2	>150
**12**	Lys-Lys-Lys-**Leu-(2-Nal)Ala-Phe-(2-Nal)Ala**	17.9	105.8	>150
**13**	(2-Nal)Ala-Phe-(2-Nal)Ala-Lys-Leu-Lys-Lys	<8	>150	>150
**14**	Lys-Lys-(*N*-Bu)Gly-Lys-(*N*1-Nal)Gly-(*N*-Phe)Gly-(*N*1-Nal)Gly	31.6	93.1	>150
**15**	Lys-Lys-Lys-(*N*-Bu)Gly-(*N*1-Nal)Gly-(*N*-Phe)Gly-(*N*1-Nal)Gly	99.5	35.61	47.6
**16**	(1-Nal)Ala-Phe-(1-Nal)Ala-Lys-Leu-Lys-Lys	11.0	145.0	>150
**17**	Lys-Lys-Lys-Leu-(2-Nal)Ala-Tyr-(2-Nal)Ala	100.1	74.7	87.7
**18**	Lys-Lys-Lys-Nle-(2-Nal)Ala-Phe-(2-Nal)Ala	98.6	64.4	87.8
**19**	Lys-Lys-Lys-Leu(1-Nal)Ala-Tyr-(1-Nal)Ala	100.6	45.0	74.9
**20**	Lys-Lys-Lys-Nle-(1-Nal)Ala-Phe-(1-Nal)Ala	<8	>150	>150
**21**	Lys-Lys-Lys-**Leu-(1-Nal)Ala-Tyr-(1-Nal)Ala**	<8	>150	>150
**22**	Lys-Lys-Lys-Nle-(2-Nal)Ala-Tyr-(2-Nal)Ala	24.2	83.0	>150
**23**	*N*-Lys-*N*-Lys-*N*-Lys-Leu-(2-Nal)Ala-Phe-(2-Nal)Ala	<8	>150	>150
**24**	Lys-Lys-Lys-**Leu-(2-Nal)Ala-Tyr-(2-Nal)Ala**	<8	>150	>150

Table S4. Haemolysis data against bovine erythrocytes

**ID**	**Sequence**	**Haemolysis 150 µM**	**IC_{10} (µM)**	**IC_{50} (µM)**
**1**	Lys-Lys-Leu-Lys-(2-Nal)Ala-Phe(2-Nal-)Ala	32.5	45.1	>150
**2**	(*N*1-Nal)Gly-(*N*-Phe)Gly-(*N*1-Nal)Gly-(*N*-Bu)Gly-Lys-Lys-Lys	<8	>150	>150
**3**	**(2-Nal)Ala-Phe-(2-Nal)Ala-**Lys-**Leu**-Lys-Lys	<8	>150	>150
**4**	**(2-Nal)Ala-Phe-(2-Nal)Ala-Leu-**Lys-Ly-Lys	<8	>150	>150
**5**	(1-Nal)Ala-Phe-(1-Nal)Ala-Leu-Lys-Lys-Lys	<8	>150	>150
**6**	Lys-Lys-Leu-Lys-(1-Nal)Ala-Phe-(1-Nal)Ala	39.9	23.6	>150
**7**	Lys-Lys-Lys-Leu-(1-Nal)Ala-Phe(1-Nal)Ala	16.0	118.8	>150
**8**	**(1-Nal)Ala-Phe-(1-Nal)Ala-**Lys-**Leu-**Lys-Lys	<8	>150	>150
**9**	**(1-Nal)Ala-Phe-(1-Nal)Ala-Leu-**Lys-Lys-Lys	<8	>150	>150
**10**	Lys-Lys-**Leu-**Lys-**(1-Nal)Ala-Phe-(1-Nal)Ala**	<8	>150	>150
**11**	Lys-Lys-Lys-**Leu-(1-Nal)Ala-Phe-(1-Nal)Ala**	17.4	89.2	>150
**12**	Lys-Lys-Lys-**Leu-(2-Nal)Ala-Phe-(2-Nal)Ala**	<8	>150	>150
**13**	(2-Nal)Ala-Phe-(2-Nal)Ala-Lys-Leu-Lys-Lys	17.6	96.7	>150
**14**	Lys-Lys-(*N*-Bu)Gly-Lys-(*N*1-Nal)Gly-(*N*-Phe)Gly-(*N*1-Nal)Gly	13.9	123.9	>150
**15**	Lys-Lys-Lys-(*N*-Bu)Gly-(*N*1-Nal)Gly-(*N*-Phe)Gly-(*N*1-Nal)Gly	<8	>150	>150
**16**	(1-Nal)Ala-Phe-(1-Nal)Ala-Lys-Leu-Lys-Lys	30.6	45.2	>150
**17**	Lys-Lys-Lys-Leu-(2-Nal)Ala-Tyr-(2-Nal)Ala	19.9	81.4	>150
**18**	Lys-Lys-Lys-Nle-(2-Nal)Ala-Phe-(2-Nal)Ala	<8	74.2	>150
**19**	Lys-Lys-Lys-Leu(1-Nal)Ala-Tyr-(1-Nal)Ala	<8	>150	>150
**20**	Lys-Lys-Lys-Nle-(1-Nal)Ala-Phe-(1-Nal)Ala	20.1	94.7	>150
**21**	Lys-Lys-Lys-**Leu-(1-Nal)Ala-Tyr-(1-Nal)Ala**	16.0	109.7	>150
**22**	Lys-Lys-Lys-Nle-(2-Nal)Ala-Tyr-(2-Nal)Ala	<8	58.4	>150
**23**	*N*-Lys-*N*-Lys-*N*-Lys-Leu-(2-Nal)Ala-Phe-(2-Nal)Ala	11.1	145.8	>150
**24**	Lys-Lys-Lys-**Leu-(2-Nal)Ala-Tyr-(2-Nal)Ala**	29.8	89.6	>150

Table S5. Cytotoxicity against HeLa, HaCaT and HepG2 cells

Table S5. Effect of selected compounds on viability of HeLa, HaCaT and HepG2 cells

ID	HaCaT IC_{50} (µM)	HepG2 IC_{50} (µM)	HeLaIC_{50} (µM)	*S.p.*[1] MIC (µM)	R.t.[2] (min)
**1**	654	n.d.	462	7.6	17.19
**2**	89	355	n.d.	7.6	17.44
**4**	433	n.d.	783	15.1	16.47
**5**	524	n.d.	1027	15.1	15.98
**11**	517	233	233	1.9	17.52
**15**	297	1481	394	3.8	17.89
**18**	n.d.[3]	n.d.	n.d.	2-4	17.46

[1]*Staphylococcus pseudintermedius*

[2]Retention time

[3]Not determined

Figure S1. Differential hydrophobicity of *in vivo* compounds

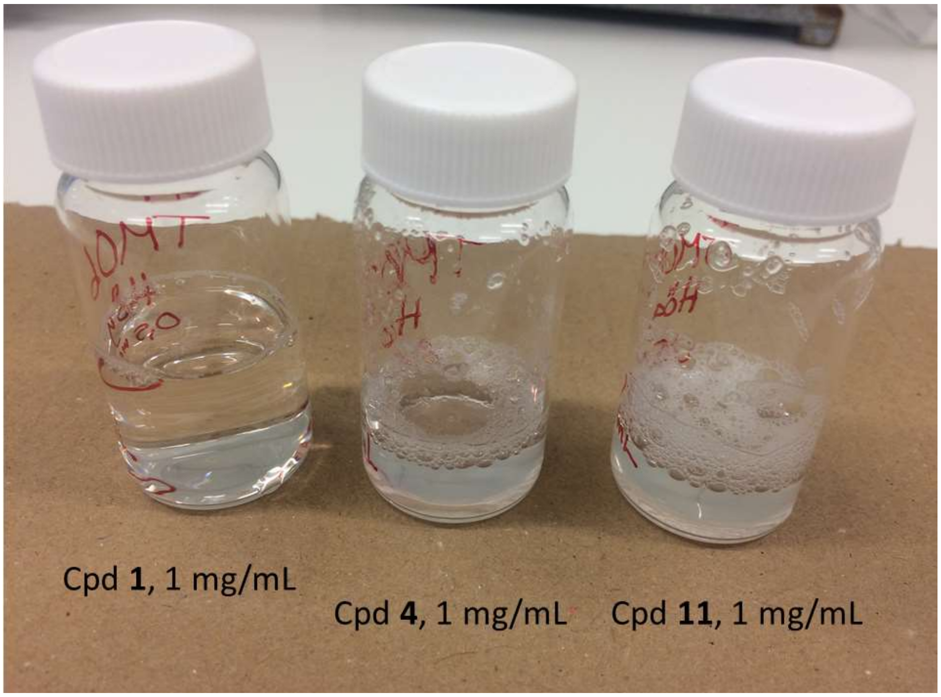

Figure S1. Illustration of hydrophobicity of the different compounds used for the *in vivo* studies in rat. Stock solutions formed very stable foams when prepared that lasted for nearly 1 h which indicates a detergent-like behaviour of the more hydrophobic compounds in PBS.

Figure S2. **Plasma concentration of urea, albumin and creatine**

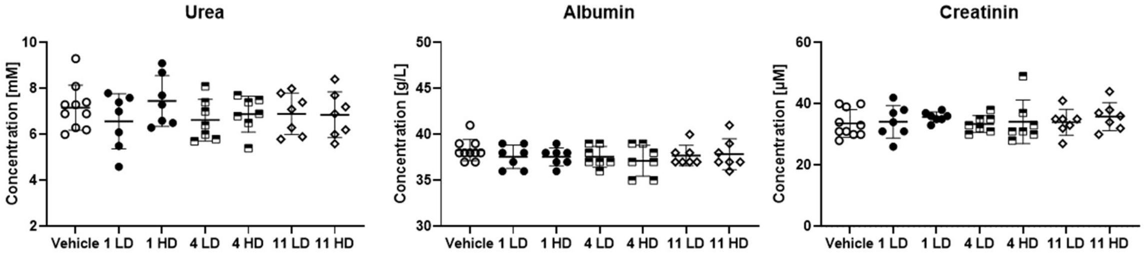

Figure S2. Plasma concentration of urea, albumin and creatine in response to injection of the peptides. No changes were observed in response.
